# Concerns About ABA-Based Intervention: An Evaluation and Recommendations

**DOI:** 10.1007/s10803-021-05137-y

**Published:** 2021-06-16

**Authors:** Justin B. Leaf, Joseph H. Cihon, Ronald Leaf, John McEachin, Nicholas Liu, Noah Russell, Lorri Unumb, Sydney Shapiro, Dara Khosrowshahi

**Affiliations:** 1 Autism Partnership Foundation, 200 Marina Drive, Seal Beach, CA 90740 USA; 2 University of California, Santa Barbara, CA USA; 3 Parent, Columbia, SC USA; 4 Parent, Seal Beach, CA USA

**Keywords:** Autism, Abuse, Behavioral intervention, Early intensive behavioral intervention, Self-stimulatory behavior

## Abstract

For over 50 years, intervention methods informed by the principles of applied behavior analysis (ABA) have been empirically researched and clinically implemented for autistics/individuals diagnosed with autism spectrum disorder (ASD). Despite the plethora of evidence for the effectiveness of ABA-based interventions, some autism rights and neurodiversity activists have expressed concerns with ABA-based interventions. Concerns have included discontent with historical events and possible harm from the procedures and goals targeted. The purpose of this manuscript is to examine some expressed concerns about ABA-based intervention and suggest productive ways of moving forward to provide the best outcomes for autistics/individuals diagnosed with ASD. The authors represent stakeholders from multiple sectors including board certified behavior analysts, licensed psychologists, parents, and autistics/individuals diagnosed with ASD.

## Concerns About ABA-Based Intervention: An Evaluation and Recommendations

Wolf and colleagues (1964) provided one of the first empirical evaluations of the application of behavior analytic principles to address the behavior of autistics/individuals diagnosed with autism spectrum disorder^1^ (ASD). In this seminal study, Wolf and colleagues examined the effectiveness of several operant conditioning procedures (e.g., extinction, shaping) to decrease the frequency of interfering behavior (e.g., tantrums) and increase the frequency of pro-social behavior (e.g., wearing glasses, bedtime behavior, communication skills) for a young autistic boy^2^ who was at risk of permanent vision loss and institutional placement. The results of the study indicated that the procedures, based on behavior analytic principles, were effective for developing a variety of skills and ameliorating interfering behavior. Furthermore, six months following the study the participant’s mother reported that her son “continues to wear his glasses, does not have tantrums, has no sleeping problems, is becoming increasingly verbal, and is a new source of joy to the members of his family” (Wolf et al., 1964, p. 312).

In the decades following Wolf et al. (1964) there have been numerous studies demonstrating the effectiveness of procedures based on behavior analytic principles for autistics/individuals diagnosed with ASD. These studies have included evaluating the effectiveness of shaping (Koegel et al., 2012), discrete trial teaching (DTT; Cihon et al., 2020), incidental teaching (McGee et al., 1985), pivotal response training (PRT; Koegel et al., 1987), naturalistic developmental behavioral interventions (NDBIs; Schreibman et al., 2015), group instruction (Ledford et al., 2008), behavioral skills training (Gunby & Rapp, 2014), functional communication training (Durand & Carr, 1991), functional analysis (Jessel et al., 2016), extinction (Hoffman & Falcomata, 2014), and response cost (Falcomata et al., 2004). Studies have also evaluated the effectiveness of comprehensive behavioral interventions for autistics/individuals diagnosed with ASD (e.g., Howard et al., 2005; Koegel et al., 1987; Leaf et al., 2011; Lovaas, 1987; Lovaas et al., 1973; Sallows & Graupner, 2005; Schreibman et al., 2015) as well as follow-ups and replications of these studies (e.g., Howard et al., 2014; Koegel et al., 1999; McEachin et al., 1993).

This substantial body of literature has led to the methods informed by applied behavior analysis (ABA) being considered evidence-based practices (National Autism Center, 2015), ABA-based interventions being widely recognized as the most effective interventions for autistics/individuals diagnosed with ASD (Smith, 2012), and endorsements from multiple organizations (e.g., Autism Speaks, The Association for Behavior Analysis International, the United States Surgeon General, National Institute of Mental Health, the American Psychological Association). It should be noted that although some communities equate the term *ABA* to DTT or Lovaas, within this paper, ABA refers to that which Baer et al., (1968, 1987) defined—one of the three branches of the science of behavior analysis (see Table 1 for descriptions and examples of terms/concepts used within this manuscript). Therefore, ABA, as a practice, refers to the application of behavior analytic principles to improve socially important behaviors, which can include multiple types of interventions (e.g., behavioral skills training, social skills groups, NDBIs).

**Table 1:** Definitions and examples of terms/concepts within this manuscript^a^

Term/Concept	Definition	Example
Applied Behavior Analysis (ABA)	One of the three branches of the science of behavior analysis. ABA, as a science, is a systematic approach to understanding behavior of social interest. As a practice, ABA is the application of behavior analytic principals to improve socially important behaviors	N/A
Aversive	An event that evokes a behavior that has terminated it in the past, punisher when presented following behavior, and/or as a reinforcer when withdrawn following behavior	When getting a ticket for speeding decreasing the likelihood of speeding in the future, we would say that speeding tickets are aversive
Behavior	Any activity of an organism does across space and time that can be counted	Walking, hitting, brushing teeth, thinking, talking, etc
Extinction	A schedule of reinforcement that involves discontinuing the reinforcement for a previously reinforced behavior that results in a decrease in the frequency of the behavior across time	When a teacher withholds attention following a child’s tantrums that previously produced the teacher’s attention
Punishment	Any context in which a response is followed by an event (i.e., stimulus change) that results in a decrease in the probability of similar responses in similar situations	When saying “No” following a student responding “four” to the question “What is 1 plus 1?” decreases the probability of responding “four” to the same question in the future
Reinforcement	Any context in which a response is followed by an event (i.e., stimulus change) that results in an increase in the probability of similar responses in similar situations	When providing access to a preferred TV show following completing homework increases the probability of homework completion in the future

^a^We refer the reader to Cooper et al. (2020) for expanded discussions of these terms/concepts as well as their uses as different parts of speech

Despite the plethora of evidence for the effectiveness of ABA-based interventions, some autism rights and neurodiversity activists have expressed concerns with the use of ABA-based interventions for autistics/individuals diagnosed with ASD (e.g., Bascom, 2014; Devita-Raeburn, 2016; Latimer, 2019; Lynch, 2019; Ram, 2020; Sequenzia, 2016). Terms and phrases such as *anti-ABA*, *ABA reform*, *dismantle* and *rebuild ABA*, and *all ABA is abuse* are common within this opposition, which can be found on social media, blog posts, non-peer reviewed journals, and peer-reviewed journals. Concerns have ranged from discontent with historical events within behavior analysis (e.g., Lynch, 2019) to current procedures and goals (e.g., Sequenzia, 2016) to *all* ABA-based interventions are abuse. The expressing of these concerns may be connected to responses from behavior analysts (e.g., Hanley, 2020), cancelation of behavior analytic conferences, petitions to change our practices (e.g., Cobbaert, n.d.), and alterations to ABA-based interventions (e.g., not addressing self-stimulatory behaviors, not attempting to improve attention and eye contact, not attempting to address cooperation).

The works of Wolf (1978), Holland (1978), Bannerman et al. (1990) and many others have illustrated the importance of evaluating the social significance of our interventions, listening to consumers’ judgements, and upholding clients’ rights and dignity. As such, concerns from consumers and those we are actively invested in helping need to be heard, reflected upon, and addressed. Given the expressed concerns about ABA-based interventions from some autism rights and neurodiversity activists, a closer examination of the some of the more commonly expressed concerns among consumers and advocates seems warranted. Therefore, the purpose of this manuscript is two-fold: 1) examine commonly expressed concerns from some autism rights and neurodiversity activists about the application of ABA-based interventions for autistics/individuals diagnosed with ASD, and 2) recommend possible pathways for behavior analysts to continually improve and progress ABA-based interventions, and, in turn, help improve the lives of autistics/individuals diagnosed with ASD and their families.

While there is no intent to dismiss or invalidate lived experiences with ABA-based interventions of autistics/individuals diagnosed with ASD, we understand that critical evaluations can sometimes be viewed through that lens. Our intention, however, is quite the opposite. We hope to provide an evaluation and discussion of expressed concerns to help determine potential pathways forward, which is why the authors of this manuscript represent stakeholders from multiple sectors including board certified behavior analysts, licensed psychologists, parents of individuals diagnosed with ASD, and autistics/individuals diagnosed with ASD. It is also important to note that all of the authors support neurodiversity, promote acceptance and accommodation, and hope that everyone would agree that autistics/individuals diagnosed with ASD have the same rights as neurotypicals. We hope this manuscript opens a dialogue between behavior analysts and those we serve about ways applied behavior analysts can proceed in the wake of the ever-increasing concerns about our science and practice.

## Concerns Over Ivar Lovaas and the UCLA Young Autism Project

Commonly expressed concerns by some autism rights and neurodiversity activists about ABA-based interventions relate directly to the research and clinical work of Ivar Lovaas and the Young Autism Project (YAP). The third and fourth authors spent collectively 20 years implementing treatment, training, supervising therapists, and conducting research with Lovaas and have written extensively to detail the history, positive and negative, of the YAP (e.g., Leaf & McEachin, 2016). They are, therefore, in a unique position to provide insight on what occurred at the YAP. Prior to the YAP, there was a widely held belief that autistics/individuals diagnosed with ASD were incapable of change and were destined to a life of institutionalization (Eikeseth, 2001). At the time there were no comprehensive intervention(s) that were found to be successful for decreasing aberrant behavior or increasing pro-social behavior for autistics/individuals diagnosed with ASD. The work by Lovaas and colleagues might appear antiquated by today’s standards and the field of ABA has surely improved since these early days. During the time period of YAP, children were literally dying or experiencing 24 h restraint to keep them from harming themselves, and many were destined to spend their entire lives in an institution (Koegel, 2015). Lovaas, however, demonstrated an approach to improve the quality of life for autistics/individuals diagnosed with ASD. Children made tremendous progress in areas such as language, social behavior, and educational goals. With this progress, institutionalization was no longer the norm or outcome for autistics/individuals diagnosed with ASD. Although ABA has certainly progressed in the past 40 years, it is still important to address the concerns about Lovaas and UCLA YAP.

### The Use of Shock

One concern about Lovaas is that he “used electric shocks to stop children from engaging in their obsessive, repetitive behaviours” (Lynch, 2019, para. 11). It is true that Lovaas used electric shock as part of the intervention in his practice at UCLA in the 1960s (pre-YAP). However, electric shock was not used for “obsessive, repetitive behaviours” (Lynch, 2019, para. 11), but for addressing life threatening self-injurious behaviors (Smith & Eikeseth, 2010). The quest for the rapid elimination of harmful behavior led Lovaas to seek procedures that allowed precise quantification of intensity and ensured brevity, which made shock the primary option at the time. By the 1970s, shock was replaced with a spank (Lovaas, 1987). The spank was implemented at the YAP in the early 1970s and was discontinued by the late 1970s. While punishment-based procedures were used, participants accessed more reinforcing than punishing consequences, and physical punishment was no longer used by the end of the YAP study (Larsson & Wright, 2011). In fact, a guiding principle of the YAP was that the ratio of reinforcement to punishment had to be at least 100:1 (Leaf & McEachin, 2016). As Rimland (1978) pointed out, “Like all behavior modification programs, his [Lovaas] was 98% positive reinforcement, with only a trace of aversive control” (p. 100).

Concerns espousing that Lovaas used physical punishment, such as shock, are accurate based on published research (e.g., Lovaas et al., 1973) and the personal experiences of those involved in his work at UCLA and the YAP. However, if those concerns are generalized to ABA-based interventions today (i.e., shock is used within modern day, progressive ABA), they lose their validity (e.g., Ram, 2020). There are no data to support that shock is commonly used within ABA-based intervention for autistics/individuals diagnosed with ASD. While there may be few examples of the contrary (e.g., the Judge Rotenberg Center), those represent exceptions and not the rule. Ultimately, it is important to acknowledge the historical uses of punishment-based procedures, why those procedures were selected, how the use of those procedures evolved over time, and ABA-based interventions, more generally, have evolved. For example, there has been an increase in the number of non-aversive/invasive alternatives to traditionally aversive/invasive procedures (e.g., Cihon et al., 2021; Ellis et al., 2006; Koegel et al., 1987; Schreibman et al., 2015). This research has demonstrated that many behaviors, once thought to only be changed through aversive/invasive procedures, can be effectively changed through non-aversive/invasive methods.

### Intervention Intensity

A second concern relates to the recommended number of hours of intervention (Latimer, 2019; Lynch, 2019). For example, “40 h a week is too much for *me* so I can’t imagine how a small child manages it” (Lynch, 2019, 40 h a week section). Concerns about children receiving 40 h a week of intervention appear to be related to misconceptions of Lovaas’ (1987) landmark study (Leaf & McEachin, 2016). That is, there appears to be a belief that participants within the YAP received exactly 40 h per week of intervention. However, intervention was not set at 40 h per week. Rather, participants received an *average* of 40 h based upon individual needs. Some received more than 40 h per week while some received much less (Lovaas, 1987).

While there is validity in that some children from the Lovaas (1987) study and within the YAP received an average of 40 h, concerns regarding this number of hours seem unfounded within the published literature. To date, there is no data to support that any number of hours of ABA-based intervention is associated with any undesired or harmful results. Furthermore, meta-analyses of outcome studies indicate that more hours of ABA-based intervention at an early age are correlated with improvements on a wide variety of measures (e.g., Eldevik et al., 2009; Roth et al., 2014; Virués-Ortega, 2010). Therefore, available data seems to indicate evidence in direct opposition of concerns related to the number of hours of intervention. It should also be noted that the average number of hours children attend school ranges from 30 to 35 h per week, which closely resembles the recommended number of hours of ABA-based intervention for autistics/individuals diagnosed with ASD.

### Rigid, Formulaic Intervention

A third concern related to Lovaas is that “Lovaas ABA was formulaic, a one-size-fits all therapy in which children for the most part started on the same lesson, no matter what their developmental age” (Devita-Raeburn, 2016, para. 26). This concern also seems to be related to misconceptions about the Lovaas (1987) study and the YAP. As Leaf and McEachin (2016) noted,

Let me assure you, it was not rigid whatsoever. You saw the film from 50 years ago. It was not rigid back then and was not rigid during our generation either. And we were certainly not protocol driven. In fact, Ivar did not believe in protocols. He wanted us to be innovative, creative, and always changing. He wanted us to probe and of course evaluate if what we were doing was effective. If it was not effective then we would change the program. “Do not adhere to protocols!” (Chance & Lovaas, 1974) Similar to an outstanding cook, you may use a recipe as a guide but be creative and improvise as you deem necessary. With the children in the study we had a structure, a plan, but were always willing, encouraged and expected to change so as to meet the needs of our children. Individualization was critical and rigid protocols were antithetical to responding to the unique and ever changing needs of the child (p. 20).

The third and fourth authors of this manuscript, as well as others, have discussed in various outlets how therapy was ever-changing, dynamic, and flexible during the YAP (e.g., Eikeseth, 2001; Larsson & Wright, 2011; Leaf & McEachin, 2016; Smith & Eikeseth, 2010). Procedures were individualized for their clients and those procedures would change moment-to-moment, which continues to be a large part of the philosophy of this progressive approach to ABA (Leaf et al., 2016).

It should be noted, however, that the repertoires and skill level across professionals in any field is likely to greatly vary. Even with minimum standards in place, this variance is likely. For example, anyone who drives is likely familiar with the varied skill level in drivers, even though a driver’s license is required to drive legally. The field of ABA, as it relates to practice, is no different. There are varied repertoires and skill levels across practicing behavior analysts that are likely to impact the quality of the intervention they provide. Training methods that produce the repertoires necessary for those providing intervention to be analysts that can “assess, adjust, and continually examine the effectiveness of their instructions” (Leaf et al., 2016, p. 722) are likely to be key. Nonetheless, continued research evaluating training methods that produce the most effective practicing behavior analysts will be helpful in improving the quality of intervention for autistics/individuals diagnosed with ASD.

### Outcomes

A final concern related to UCLA YAP is the outcomes of Lovaas (1987) and behavior analysts trying to achieve similar outcomes today. In a paper evaluating the line between intervention and abuse, Kirkham (2017) illustrated the feelings of some in the neurodiversity movement about the outcomes of ABA-based intervention. For example, Kirkman stated “Prominent self-advocate Amy Sequenzia has similarly criticized ABA for unjustifiably holding up neurotypical behaviour as an ideal, arguing that its attempt to ‘change how an autistic acts, reacts or interacts with the world’ is wrong” (p. 117). Kirkman further stated “Amanda Vivian (2012) has concluded that it is wrong to say ‘cure autism now’” (p. 117).

There have been many different terms within the research on early intensive behavioral intervention (EIBI) to describe outcomes for autistics/individuals diagnosed with ASD including cure (e.g., Lovaas et al., 1973), recovery (e.g., Stubbs et al., 1976), best outcome (McEachin et al., 1993), and indistinguishable (McEachin et al., 1993). These terms were used to represent a subgroup of autistics/individuals diagnosed with ASD who, after receiving EIBI, no longer met the diagnostic criteria for ASD. The term “cure” is a particularly problematic term, because it implies that the cause of a disorder has been identified and removed. Even in the 1970s, Lovaas disavowed the notion of cure. “Recovery” may have seemed like a more appropriate term, but may still be problematic because of the negative connotations associated with recovery. These terms are only as meaningful as the definition that is attached to them using objective, measurable criteria. Of note is that researchers did not rely on subjective self-reports, but, rather, objective and standardized measures such as IQ scores (in the normal range). Additionally, the measures included placement (e.g., general education classrooms) and behaviors associated with the Diagnostic and Statistical Manual of Mental Disorders (American Psychiatric Association, 2013). There are some who have invoked the concept of masking as described by Ekman (1972), claiming that all individuals diagnosed with ASD learn to mask their behavior to conform to societal norms but remain essentially autistic. However, given measures of the outcomes of EIBI are standardized and objective, it is difficult to support the claim of masking. Further, regardless of the terminology used, it is probable that increasing cognition (Harris et al., 1991), language (Smith et al., 2000), play (Ben-Itzchak & Zachor, 2007), social behavior (Lovaas et al., 1973), and adaptive behavior (Anderson et al., 1987), while decreasing aberrant behavior (Lovaas et al., 1973) improves quality of life through the development of repertoires that empower and enhance options.

### Recommendations

There are several potential pathways forward with respect to concerns related to the research and clinical work of Ivar Lovaas and the YAP. First, continued development of practicing behavior analysts’ knowledge of past research, including that of Lovaas, will permit more accurate identification of strengths, weaknesses, positives, and mistakes within this research. This will, in turn, permit continual evolution, improvements, and refinements of the methods informed by our science. Second, it is imperative that practicing behavior analysts continue to implement and advocate positive reinforcement-based contingencies when possible while designing interventions for autistics/individuals diagnosed with ASD. Which will hopefully lead to members of the neurodiversity movement and other professionals to be more accepting of ABA-based interventions.

Third, the intensity of intervention should be determined at the individual level. Pre-determined or automatically recommending 40 h per week based on averages found in the literature should be avoided. It should be noted, however, that meta-analyses have found that more hours of ABA-based intervention at an early age are correlated with improvements (e.g., Eldevik et al., 2009; Roth et al., 2014; Virués-Ortega, 2010). Nonetheless, intensity of intervention should be individualized and frequently assessed for responsiveness to intervention, affect, and consumer happiness. Future research should evaluate variables associated with the relationship between demographics and intensity as well as ancillary measures associated with intensity (e.g., long-term outcome data, consumer judgements across time).

Fourth, behavior analysts should continue to evolve and progress the methods informed by our science. This progression should include a move away from rigidly adhering to protocols and a move toward the use of in-the-moment analysis in more naturalistic contexts. That is not to say that since the time of the UCLA YAP, the methods informed by our science have not evolved or progressed—quite the contrary. For example, PRT is one intervention type based on behavior analysis that uses natural instructions and materials to optimize instruction for autistics/individuals diagnosed with ASD (Koegel et al., 1987). There has been a plethora of experimental studies which have demonstrated the effectiveness of PRT (Koegel et al., 1999). Another example of the evolution of behavioral intervention comes in the form of NDBIs (Schreibman et al., 2015). NDBIs combine best-practices from developmental science and ABA to promote engagement, social motivation, and synchrony between the parent and child, while using operant learning strategies to teach specific skills. Finally, Progressive ABA is an approach in which interventionist behavior is controlled by in-the-moment assessment of environmental variables (e.g., client affect, past client performance), sometimes referred to as clinical judgement (Leaf, Leaf, et al., 2018a, 2018b). Ultimately, the goal of a progressive approach to ABA is to consistently evolve and progress our methods and outcomes which should continue to be a focus for all interventions and methods informed by behavior analysis.

## Punishment- and Extinction-based Procedures

### Punishment

Some autism rights and neurodiversity activists have expressed that the use of punishment-based procedures within ABA-based interventions (e.g., Devita-Raeburn, 2016; Ram, 2020) is inhumane and harmful. For example, Ram (2020) posed the question “In what world is it okay to attach a shock device to someone and give the power to shock them to other humans?” (Judge Rotenberg Center section). It is, perhaps, unfortunate that our field adopted the term *punishment*, because to the general public punishment has numerous connotations, including retribution (i.e., “an eye for an eye”) and pain. As such, it is important to preface this discussion that behavior analysts define punishment differently than the general public. Punishment, from a behavior analytic perspective, describes any context in which a response is followed by an event (i.e., stimulus change) that results in a decrease in the probability of similar responses in similar situations. Punishment, just like reinforcement, is a naturally occurring principle of behavior. As Vollmer (2002) noted, “punishment occurs like the wind and the rain” (p. 469). Absent from this definition are things like pain, fear, discomfort, and the like. Suppose a person parks their car taking up two spaces and a passerby comments, “That’s inconsiderate.” If the probability of taking up two spaces while parking subsequently decreases, we can reasonably presume that punishment occurred. This is not to say that examples of punishment cannot, or do not, include situations in which pain or discomfort occur such as Ram’s example of the use of shock.

The field of ABA has had a precarious history with the use and research of punishment-based procedures (Baer, 1970; Dinsmoor, 1977; Horner, 2002; Lerman & Vorndran, 2002; Miltenberger, 2001; Vollmer, 2002). Early in the history of ABA, a variety of punishment-based procedures were evaluated and implemented including electric shock (Risley, 1968), water misting (Dorsey et al., 1980), spankings (Foxx & Azrin, 1973), and restriction of movement (Green & Striefel, 1988). Since those early days, for the most part, practitioners have decreased the reliance on using such procedures in favor of more preferred, reinforcement-based procedures. In fact, the Professional and Ethical Compliance Code for Behavior Analysts requires exhausting the use of reinforcement procedures prior to the use of punishment-based procedures (Behavior Analyst Certification Board, 2014). Some agencies and clinicians refuse to implement any punishment-based procedures while other agencies still implement non-invasive punishment-based procedures (e.g., saying “No, not that is not it, try again” following an incorrect response, removal of preferred items, time-out; Leaf et al., 2019). Yet, even today, some agencies still implement more invasive punishment-based procedures (e.g., electric shock; Blenkush, 2017). Even Positive Behavior Support guidelines included the use of strong aversives under some circumstances (Brown et al., 2008), and Electric Convulsive Therapy (ECT) is often the recommended intervention with severe depression (The UK ECT Review Group, 2003).

While research has demonstrated the effectiveness of punishment-based procedures in reducing the likelihood of similar behavior occurring (adaptive or aberrant), many have associated punishment, more generally, with occasioning undesired side effects (Lerman & Vorndran, 2002; Risley, 1968). In their extensive review of basic and applied findings related to punishment, Lerman and Vorndran (2002) noted that textbooks and literature reviews commonly discuss aggression, escape behavior, and emotional reactions among these side effects. However, Lerman and Vorndran also noted that applied research has demonstrated a variety of desirable effects from the use of punishment-based procedures. Nonetheless, the possibility of side effects is likely a reason some autism rights and neurodiversity activists have expressed opposition to the use of any punishment-based procedures.

Based upon the literature, there is some validity to concerns with the use of punishment-based procedures for autistics/individuals diagnosed with ASD. That is, if punishment-based procedures have been documented to result in undesired side effects, there may be cause for concern with the use of those punishment-based procedures within the same conditions. However, as Lerman and Vorndran (2002) noted, “The prevalence of these side effects is unknown, however, because relatively few studies have directly examined the effects of punishment on unpunished behavior in clinical settings” (p. 454). As such, to fully evaluate concerns of the use of punishment-based procedures for autistics/individuals diagnosed with ASD, more research is necessary. This research could help to (1) examine if there are differences in possible side effects when comparing more and less invasive punishment-based procedures (e.g., saying “no” for incorrect responses in comparison to time out from positive reinforcement; see Leaf et al., 2019 for an example), (2) identify if there are conditions under which more invasive punishment-based procedures may be necessary, (3) prevent the misuse of punishment-based procedures under the guise of ABA-based intervention, and (4) inform effective reinforcement-based alternatives.

### Extinction

Concerns about ABA-based procedures are not limited to punishment-based procedures, as some autism rights and neurodiversity activists have expressed concerns with the use of extinction procedures (e.g., Ram, 2020). Ram (2020) noted, “Extinction (including planned ignoring) goes against what all the research is showing us about child and human development. Extinction doesn’t care about trauma, in fact it can cause trauma” (use of punishment, extinction, shock section). Ram did not expand on how extinction is at odds with research on child and human development, making it difficult to further examine this claim. It is possible, however, to further examine the claim that extinction causes trauma. Similar to the aforementioned discussion surrounding punishment, it is important to note that behavior analysts define extinction functionally as termination of a response-reinforcer contingency, which is commonly done through non-delivery of a previously delivered reinforcer. Said differently, extinction involves withholding a reinforcer contingent upon a response that previously resulted in access to the same reinforcer.

Procedures involving the use of extinction are, perhaps, more prevalent in the literature addressing self-injurious behavior, aggression, and pediatric feeding disorders. Over 30 years ago Lerman et al. (1999) analyzed 41 data sets of individuals who received treatment for self-injurious behavior that included extinction for possible side effects (i.e., extinction bursts or aggression). Lerman et al. found that 15 of the 30 participants showed extinction bursts or aggression, and that the occurrence of these side effects was mitigated when extinction was combined with differential reinforcement. While there was no mention of trauma, Lerman and colleagues were only evaluating the literature for the occurrence of extinction bursts or aggression, and the authors of this manuscript are unaware of any extensive literature reviews on the use of extinction and trauma. In fact, many studies evaluating procedures with an extinction component have documented favorable outcomes. For example, Grow et al. (2008) found that extinction within functional communication training promoted response variability to identify a response to reinforce as an alternative to problem behavior. Relatedly, Hanley et al. (2005) found that both children with severe behavior disorders who participated in their study preferred the functional communication training (which included an extinction component) condition that also included a punishment contingency for problem behavior. Piazza et al. (2003) found that reinforcement alone was less effective when compared to reinforcement plus escape extinction with four children diagnosed with a pediatric feeding disorder. Furthermore, positive reinforcement combined with escape extinction reduced extinction bursts, inappropriate behavior, and crying for some participants (Piazza et al., 2003).

Currently, the research evaluating procedures involving extinction do not seem to provide evidence that extinction results in trauma. However, most literature reviews and studies evaluating procedures involving extinction did not include direct evaluations of possible trauma. As such, future research will be necessary to fully evaluate claims of extinction induced trauma. This could take the form of literature reviews of studies examining the effectiveness of procedures involving extinction and scanning for any mention or indication of trauma. Research could also explicitly evaluate conditions that do or do not include and extinction component while evaluating participant preference and measures of trauma. Ultimately this research is likely to identify the conditions under which procedures involving extinction are appropriate and necessary. For instance, if an otherwise healthy child engages in food refusal to the point of malnourishment, escape-extinction may be, at least initially, necessary. While if an otherwise healthy child engages in food selectivity, it may be appropriate to avoid the use of escape-extinction altogether (Riordan et al., 1980).

### Recommendations

While awaiting the results of more research on punishment and extinction, an approach behavior analysts might take to help address any confusion and concerns is to have meaningful discussions with stakeholders and autistics/individuals diagnosed with ASD. These discussions may benefit from including a behavior analytic conceptualization of punishment (i.e., a functional relationship) and how that contrasts with traditional conceptualizations of punishment. Hopefully these discussions can be informed by our field’s history related to the use of punishment- and extinction-based procedures and the most current and relevant research. As such, blanket statements from behavior analysts that all punishment-based procedures are to be abandoned should be avoided within these discussions as they do not align with the research or take into consideration the functional definition of punishment and the naturally occurring behavioral phenomenon that is punishment. This is not to say that others within these discussions (e.g., autistics/individuals diagnosed with ASD) could not make similar statements, as these should open discussions in an attempt to listen and learn. Including several professionals, organizations (e.g., CASP, ABAI, APBA), and autistics/individuals diagnosed with ASD could lead to the development of guidelines about the conditions under which various punishment- and extinction-based procedures are acceptable and necessary as well as whether there are any punishment- and extinction-based procedures that are never acceptable or necessary. These guidelines could then be reflected in ethical codes for certified and licensed behavior analysts.

## Self-Stimulatory and Stereotypic Behavior

Some autism rights and neurodiversity activists have expressed concerns with addressing stereotypic behavior (sometimes referred to as “stimming”) within ABA-based intervention. Examples have included, “stimming helps decrease anxiety and stress in autistics” (Ram, 2020, Masking section) and “stimming is a comforting self-soothing behavior which helps us reduce stress, feel more comfortable in uncomfortable environments, and regulate our emotions” (Lynch, 2019, ABA is not designed section). Furthermore, surveys of autistic adults “reported [stimming] to be a useful behaviour, serving to contain or control excess emotion” (Kapp et al., 2019, p. 1788). As such, concerns about ABA-based interventions addressing stereotypic behavior often involve claims that behavior analysts do not understand why autistics/individuals diagnosed with ASD engage in stereotypic behavior (Fahrenheit, 2020), society should be accepting of stereotypic behavior (Kapp et al., 2019), and targeting stereotypic behavior is abusive (Fahrenheit, 2020).

Behavior analysts view stereotypic behavior as functionally related to observable environmental variables. As a result, through a behavior analytic lens, stereotypic behavior is a product of its circumstances. Behavior analytic research supports the common presumption that stereotypic behavior serves an automatic or non-social function; however, research has also shown stereotypic behavior to be multiply determined and the result of positive social reinforcement and escape and avoidance contingencies (Cunningham & Schreibman, 2008). World views that offer competing or alternative explanations for behavior are likely to result in concerns such as behavior analysts not understanding why autistics/individuals diagnosed with ASD engage in stereotypic behavior.

It is our hope that all behavior analysts support building a more inclusive world where people are more accepting of differences, behaviorally or otherwise. However, behavior analysts also have an obligation to best prepare their clients for the world in which they currently live, which is, unfortunately, less accepting than desired. Research has documented that engaging in stereotypic behavior often has a negative impact on the person engaging in the stereotypic behavior (Bodfish et al., 2000; Goldman et al., 2009; Koegel et al., 1974) as well as negative perceptions from those observing the person engaging in the stereotypic behavior (Cook & Rapp, 2020a; Welsh et al., 2019). To align with the research and best prepare clients for the environments in which they will find themselves, behavior analysts will likely be required to address stereotypic behavior. However, ABA-based interventions should work toward empowering and enhancing options rather than achieving conformity. Developing the repertoires necessary to choose whether or not to adapt to different situations based on an analysis of the possible outcomes.

### Recommendations

All claims of abuse should be taken seriously and claims that targeting stereotypic behavior is abusive are no different. If a person indicates they were abused, they should take all possible actions in accordance with local and federal laws. With respect to the purpose of this manuscript, of importance is evidence of abuse within the research on interventions addressing stereotypic behavior. Literature reviews indicate that “reducing stereotypy generally leads to [desirable] changes in other behaviors” (Lanovaz et al., 2013, p. 1240) such as item engagement (Zhou & Goff, 2000), vocalizations (Celiberti et al., 1997), communication (Anderson et al., 2010), sitting (Lanovaz et al., 2013), play (Bennett et al., 2011), correct responding (Rosenthal-Malek & Mitchell, 1997), and academic tasks (Cook & Rapp, 2020b). None of the reviews of studies that have evaluated interventions for addressing stereotypic behavior (e.g., Akers et al., 2020; Chebli et al., 2016; DiGennaro-Reed et al., 2012; Lanovaz et al., 2013; Rapp & Vollmer, 2005; Wang et al., 2020) have indicated harm or abuse experienced by participants. While not discounting others lived experiences, it seems as though participants of studies evaluating interventions to address stereotypic behavior have not made these claims within the research. Nonetheless, more research is necessary to fully evaluate claims of harm, trauma, and abuse as a result of ABA-based interventions addressing stereotypic behavior. This research could include continued evaluation of the conditions under which stereotypic behavior occurs as well as what forms may interfere with the development of friendships, obtaining and maintaining employment, and preventing others from working and learning in the same environment. This research will be essential in identifying if some forms of stereotypic behavior may be best to engage in while others are not present as not to decrease opportunities, better understand which forms of stereotypic behavior are more or less socially acceptable, and how we can teach society to be more understanding and tolerant of stereotypic behavior.

## Goal Selection and the Goals of Therapy

Expressed concerns about goal selection within ABA-based intervention are multi-faceted. They have included that autistics/individuals diagnosed with ASD are not included in the goal selection process, selected goals are inappropriate (e.g., eye contact), and ABA-based interventions fundamentally change the individuality and personality of the client (Devita-Raeburn, 2016; Lynch, 2019; Ram, 2020; Sequenzia, 2016). For example, Lynch (2019) noted, “Most ABA therapists don’t set out to hurt children. And yet, despite making ABA therapy fun and positive, the underlying goals of ABA have not changed. And it is these goals that, like gay conversion therapy, do long-term damage to the human psyche” (15^th^ paragraph) and “They [behavior analysts] don’t see how weird it is to try systematically to shape a child’s behaviour to teach them to play with a toy the ‘right’ way” (But I do know what autism feels like section). In another example Sequenzia, 2016 claimed that, “Because ABA proponents, as their philosophy dictates, never accept a ‘no’ from Autistics, and will relentless pursue the goal of making us comply with what they believe is the desirable way of being, their next argument was to say that ‘neurotypical people comply all the time’, [sic] that’s why it is vital that Autistics learn compliance too” (11^th^ paragraph).

### Goal Selection Process and Selected Goals

Early in the conceptualization of the ABA, Baer et al. (1968) noted that the goals within ABA research should be of importance to society; a point expanded upon by Baer et al. (1987). Wolf (1978) further contended that behavior analysts should actively seek consumer evaluations of the acceptability of their goals, procedures, and results. Collectively, Wolf referred to this as *social validity*, which has long been a hallmark of ABA. In fact, this is so central to ABA-based interventions that it is included in the Professional and Ethical Compliance Code for Behavior Analysts (Behavior Analyst Certification Board, 2014). Several reviews of the inclusion of social validity measures within behavior analytic research have been published (i.e., Carr et al., 1999; Ferguson et al., 2019; Kennedy, 1992). Based upon these reviews, there is validity to the concern that autistics/individuals diagnosed with ASD are not commonly included in the goal selection process, at least within the published literature. Equally concerning is the lack of improvements in the reporting of social validity measures within the research across these reviews. What remains unclear is if the results of literature reviews on the inclusion of social validity measures is reflective of clinical, home, and community settings in which ABA-based intervention commonly occurs.

### Changes to Individuality and Personality

It is important to preface this section with a discussion of personality through a behavior analytic lens. Skinner (1974) described personality as a way to describe “…a repertoire of behavior imparted by an organized set of contingencies” (p. 164). From this perspective, then, *personality* is simply a term used to describe commonly displayed patterns of behavior. As such, behavior is not attributed to or caused by a personality, as behavior occurs independent of a description of personality. If viewed through a behavior analytic lens, through which changes in behavior do not equate changes in personality, then there is little validity the claim that ABA-based intervention fundamentally changes the personality of autistics/individuals diagnosed with ASD. While the goal of ABA-based intervention for autistics/individuals diagnosed with ASD is to develop repertoires that empower and enhance options, some will likely continue to view their behaviors or patterns of behavior as part of their identity. In these situations, those individuals are likely to view any intervention that changes behavior as a threat to their identity, behavior analytic or otherwise. These are likely to be most difficult situations for behavior analysts to navigate when confronted with these concerns.

The behavior analytic view of personality differs from traditional views in which behavior is said to be caused by personalities. For instance, a delinquent child acts out due to a disorderly personality or an adult avoids social situations due to an anti-social personality. From a more traditional view of personality and behavior, there is validity to the concern that ABA-based intervention fundamentally changes the personality of autistics/individuals diagnosed with ASD. This view purports that any change in behavior would, in turn, be a change to one’s personality. It is probable that this traditional view is related to concerns from autism rights and neurodiversity activists about ABA-based intervention fundamentally changing the personality of autistics/individuals diagnosed with ASD.

It is also important to note that ABA-based intervention involves teaching language so that a child may communicate their desires, express their affection to their parents, communicate with their peers or colleagues, self-advocate, and express their displeasure (e.g., “I don’t want to do that,” “I’m uncomfortable with that”). Social behaviors are targeted because doing so increases the opportunity for friendships, dating, collaboration with others (Bauminger & Kasari, 2000), or just getting along with others in the workplace or community. Doing so also decreases the likelihood of potential negative outcomes such as loneliness, depression, and suicide (Bauminger & Kasari, 2000). Targets such as imitation, receptive instructions, and sitting better prepare clients to learn on their own and have the foundational skills to become competent learners and accomplish more complex skills like reading, cooking, and balancing a checkbook. ABA-based intervention sometimes involves exposing clients to situations that require waiting, doing something in a different way, and tolerating disappointment because we know our clients are capable of developing those skills. Individuals who can tolerate adversity are better prepared to survive in an imperfect world and will have more opportunities for life-enriching experiences. These skills permit successfully navigating societal standards and rules, and to understand how one’s behavior might be an obstacle to achieving their goals. In this sense, behavior analysts are no different than teachers in general education settings, psychologists who work with clients who have paralyzing phobia, depression, or an unhappy marriage, nutritionists who help people maintain better health, or parents who teach their children right from wrong. The main goal across each of these is to teach skills that will lead to improvements in an individual’s life. Behavior analysts, like any other helping professional, should be teaching skills that will be valuable to their clients.

### Recommendations

Simply put, practicing behavior analysts must include clients when possible, or their proxy when not, in goal selection. If a client is too young or does not have well developed communication repertoires to articulate preferences related to goal selection, then the client’s caregivers/guardians should be involved in the goal-selection process. In situations in which communication repertoires may make obtaining consent directly from our clients difficult, other methods of assent may be employed (e.g., concurrent chains; Hanley, 2010). This does not, however, mean that only the goals that clients or caregivers identify as important are targeted or not, or that only the goals that the behavior analyst identifies as important are targeted or not. For instance, if a client was only interested in improving their videogame skills at the expense of improving other necessary adaptive skills, it may be beneficial to work with that client on the identification of other meaningful goals. As another example, a behavior analyst should not select goals solely because it will check off a box on a standardized assessment. Ultimately, reasonable efforts must be taken to ensure the goal selection is a collaborative process whenever possible.

It is important to note that the training behavior analysts complete prior to working as supervisors or obtaining certification involves, or should involve, the development of repertoires related to identifying meaningful, functional, adaptive, and developmentally appropriate curricula. As such, a behavior analyst’s level of expertise for determining goals for intervention should not be discounted. However, there may be situations in which clients and/or guardians disagree with a behavior analyst’s recommendation. In these situations, possible courses of action for the behavior analyst may include (a) listening to the client’s and/or guardian’s rationale for the disagreement, (b) discussing with the client and/or guardians why the proposed goals are important in the short and long term, (c) collaborating with the client and/or guardians to identify goals everyone agrees upon, (d) working to inform consumers about the activities and learning objectives that are correlated with quality outcomes, and, if necessary, (e) providing referrals to other service providers that may be more appropriate if unable to come to a mutual agreement on the selected goals.

Ultimately, including clients in the goal selection process, when possible, could assist researchers and clinicians in identifying which goals may be viewed as not socially valid to which individuals. It may be the case that some goals have been deemed socially acceptable by those receiving ABA-based interventions and/or their caregivers, but not those outside of the intervention context (e.g., some autism rights and neurodiversity activists). These discussions could assist in how to approach these disagreements and pathways forward. Relatedly, these discussions could assist in ensuring behavior analysts teach skills that are functional, applied, and meaningful for their clients. Ultimately, these discussions could lead to more meaningful goals being targeted more frequently that enhance choices and improve the quality of life of our clients.

Practicing behavior analysts must take active steps to live up to Wolf’s (1978) vision of social validity. This would mean assessing the social validity of goals in research as well as practice with a variety of consumer judges at each level of selection. Peer reviewed journals which publish studies on behavior analysis that relates to autistic/individuals diagnosed with ASD (e.g., *Journal of Applied Behavior Analysis*, *Education and Training in Autism and Developmental Disabilities, or the Journal of Autism and Developmental Disorders)* could assist in this endeavor by requiring measures of social validity prior to acceptance for publication. Graduate training programs, which commonly develop repertoires related to conducting and reporting research, should include explicit instruction on the assessment of social validity within research. The assessment of the social validity of goals within practice may already be frequently occurring, but there are no mechanisms in place for the reporting of social validity data in practice. It is possible that one method of assessing social validity in practice is reflected in retaining clients and obtaining new clients. Nonetheless, researchers could survey owners and employees of ABA-based service providers to identify practices related to the assessment of social validity to help in this endeavor.

## Abuse and Long-Term Negative Outcomes

The final commonly voiced concern it is that ABA-based interventions and/or specific ABA-based procedures are abusive and lead to serious negative outcomes such as depression, anxiety, and/or post-traumatic stress disorder (PTSD; Kupferstein, 2018). This is evident by comments such as, “…children subjected to ABA have PTSD symptoms at a statistically higher rate than autistic people who had not had ABA” (Latimer, 2019, first paragraph), “ABA for autism is institutional abuse…” (Cobbaert, n. d., first paragraph), and “These advocates, many of them childhood recipients of ABA, say that the therapy is harmful” (Devita-Raeburn, 2016, 8^th^ paragraph). As previously stated, all claims of abuse should be taken seriously and evaluated thoroughly. There are two notable examples of peer-reviewed publications that appear to provide support for claims that ABA-based interventions are abusive or cause PTSD.

First, Kupferstein (2018), published in *Advances in Autism*, surveyed 460 respondents in an effort to evaluate a correlation between receiving and/or having received ABA-based intervention and the prevalence of posttraumatic stress symptoms (PTSS). This correlation was evaluated using a self-designed questionnaire. Kupferstein found that 46% of respondents met the diagnostic threshold for PTSD after ABA-based interventions. This number was also higher than those respondents receiving interventions with limited to no empirical base (e.g., Rapid Prompting Method, DIR/Floortime, Facilitated Communication). Second, Sandoval-Norton and Shkedy (2019), published an article entitled “How much compliance is too much compliance; is long-term ABA therapy abuse?” in *Cogent Psychology*. In this article, Sandoval-Norton et al. criticized the discipline and practice of ABA with accusations of unethical behavior, ineffectiveness, promoting learned helplessness, destruction of internal motivation, and psychological abuse and trauma. Ultimately, Sandoval-Norton and Shkedy came to the conclusion that “These children are the population that was chosen to be the subjects of an experimentally intense, lifelong treatment within a therapy where most practitioners are ignorant regarding the Autistic brain—categorically, this cannot be called anything except abuse” (p. 6).

Taken together, Kupferstein (2018) and Sandoval-Norton and Shkedy (2019) appear to provide validity to expressed concerns of abuse within ABA-based interventions. However, these articles have also been critically evaluated within the same journals. Sepcifically, Leaf, Ross, et al., (2018a, 2018b) evaluated the methodology and Kupferstein’s discussion of their results and Gorycki et al. (2020) provided an analysis and response to the claims made by Sandoval-Norton and Shkedy. Leaf, Ross, et al. concluded “that service providers, behavior analysts, funding agencies, and parents should carefully and objectively evaluate this study [Kupferstein (2018)] prior to avoiding making recommendations for ABA-based interventions for individuals diagnosed with ASD based upon the results” (p. 127). Gorycki et al. concluded that “Many of their [Sandoval-Norton and Shkedy’s] arguments are based on published reports for which there is little reliability or replication, with no connection to ASD or ABA, with literature existing that contradicts the claims made by Sandoval-North and Shkedy, but is conveniently ignored by them” (p. 9).

### Recommendations

Based upon these evaluations, behavior analysts should remain compassionately skeptical when confronted with generalizations and broad statements that ABA is abusive (e.g., Latimer, 2019). This means showing compassion, listening and learning from lived experiences, and, if applicable, referring to appropriate services (e.g., psychological help). This does not mean negating lived experiences or the substantial evidence that supports the use of ABA-based interventions for autistics/individuals diagnosed with ASD. Currently there is a lack of reliable data and research that ABA-based interventions have resulted in a diagnosis of PTSD, anxiety, or depression. Well-designed research will be vital to know the characteristics, if any, of ABA-based interventions that might have led to these outcomes. We encourage researchers from the field of behavior analysis to work collaboratively with researchers from other fields (e.g., psychology, education, research methodology) and autistics/individuals diagnosed with ASD to design methodologically sound studies on the long term effects of ABA-based interventions with respect to PTSD, anxiety, or depression. Additionally, we should evaluate the positives of behavioral intervention (e.g., happiness or restrictiveness of living arrangements) This research should involve behavior analytic service providers and organizations representing service providers (e.g., CASP) recruiting a large number of autistics/individuals diagnosed with ASD who have received services and who are now adolescents or adults. To prevent bias, evaluators should be kept blind to the purpose of these studies and should not include behavior analysts. These studies should also be authored by individuals other than behavior analysts to prevent any perceived conflicts of interest. Developing and expanding this body of research will provide a clearer picture of the prevalence of PTSD as a result of ABA-based interventions which will, in turn, inform large-scale changes in ABA-based interventions.

Outcome measures used to assess the effectiveness of comprehensive ABA-based interventions in practice and research should also be expanded. Behavior analysts should include a variety of standardized assessments across a variety of domains as measured through intelligent quotient tests, the Vineland Adaptive Behavior Scales (Sparrow et al., 2016), Expressive One Word Picture Vocabulary test (Martin & Brownell, 2011), Peabody Picture Vocabulary test (Dunn & Dunn, 2007), Aberrant Behavior Checklist (Aman et al., 1985), Social Skills Improvement System (Gresham & Elliott, 2008), Social Responsiveness Scale (Constantino, 2002), Parenting Stress Index (Abidin, 1990), and the Gilliam Autism Rating Scale (Gilliam, 2014) within research as well as clinical settings. This will permit the assessment of progress and outcomes across settings as well as overtime. In addition to the standardized assessment of desired outcomes, researchers and clinicians should use standardized assessments of undesired outcomes such as the Spence Children’s Anxiety Scale (Spence, 1997). It should be noted, however, that many standardized assessments of constructs such as anxiety or trauma have not been normed with autistics/individuals diagnosed with ASD. Additionally, researchers and clinicians should include quality of life measures such as affect (e.g., Koegel et al., 2009), happiness (Thomas, Charlop, Lim, & Gumaer, 2021) and the development of social networks and friendships (Kasari et al., 2011).

## Conclusion

In whichever area methodologies based upon the science of behavior analysis have been applied, improvements have occurred (cf. Friman, 2021). This is exemplified in the thousands of studies that have demonstrated positive outcomes of ABA-based interventions and procedures for autistics/individuals diagnosed with ASD. Despite these positive outcomes, concerns with the use of ABA-based interventions have been expressed by autism rights and neurodiversity activists in multiple outlets and some board certified behavior analysts have joined the discussion (e.g., Ram, 2020). Evaluating and discussing these concerns within the peer-reviewed literature provides an opportunity to identify potential solutions so the field can proceed in a productive, collaborative, and sensitive manner with the community for whom we are fortunate enough to serve. Based on our review of the concerns highlighted within this manuscript in light of the published literature, there is some validity to some of these expressed concerns (e.g., the collection of social validity measures in the published research) and limited to no validity to others (e.g., all ABA is abuse). Nonetheless, our field is not infallible, and we should continue to improve and progress our interventions. As Baer et al. (1968) so eloquently asserted, the continued examination of behavior analytic applications to solve problems of social significance will help assist in their refinement and, possibly, their replacement by better applications (p. 91).

## Footnotes

1 This terminology was selected to adhere to the 7^th^ edition of the American Psychological Association Publication Manual and to be inclusive of those who prefer person-first as well as identity-first language.

2 When discussing prior research, the terminology used within that research is used.
